# Tetra-gel enables superior accuracy in combined super-resolution imaging and expansion microscopy

**DOI:** 10.1038/s41598-021-96258-y

**Published:** 2021-08-20

**Authors:** Hsuan Lee, Chih-Chieh Yu, Edward S. Boyden, Xiaowei Zhuang, Pallav Kosuri

**Affiliations:** 1 Howard Hughes Medical Institute, Harvard University, Cambridge, MA USA; 2 Department of Chemistry and Chemical Biology, Harvard University, Cambridge, MA USA; 3 Department of Physics, Harvard University, Cambridge, MA USA; 4 McGovern Institute for Brain Research, MIT, Cambridge, MA USA; 5 Howard Hughes Medical Institute, MIT, Cambridge, MA USA; 6 Department of Biological Engineering, MIT, Cambridge, MA USA; 7 Department of Brain and Cognitive Sciences, MIT, Cambridge, MA USA; 8 Present Address: Salk Institute for Biological Studies, La Jolla, CA USA

**Keywords:** Super-resolution microscopy, Molecular imaging

## Abstract

The accuracy of expansion microscopy (ExM) depends on the structural preservation of samples embedded in a hydrogel. However, it has been unknown to what extent gel embedding alters the molecular positions of individual labeled sites. Here, we quantified the accuracy of gel embedding by using stochastic optical reconstruction microscopy (STORM) to image DNA origami with well-defined structures. We found that embedding in hydrogels based on polyacrylamide, the most widely used chemistry in ExM, resulted in random displacements of labeled sites with a standard deviation of ~ 16 nm. In contrast, we found that embedding in tetra-gel, a hydrogel that does not depend on free-radical chain-growth polymerization, preserved labeled sites with a standard deviation of less than 5 nm. By combining tetra-gel ExM with STORM, we were able to resolve 11-nm structural features without the loss in accuracy seen with polyacrylamide gels. Our study thus provides direct measurements of the single-molecule distortions resulting from hydrogel embedding, and presents a way to improve super-resolution microscopy through combination with tetra-gel ExM.

## Introduction

Super-resolution optical microscopy methods^1^ have made it possible to investigate structural features on length scales smaller than the diffraction limit of light. Combination of these methods with expansion microscopy (ExM)^2^ holds the potential to improve imaging capabilities further, through embedding the sample in a hydrogel followed by physical expansion of the gel. Studies combining ExM with structured illumination microscopy (SIM)^3,4^, stimulated emission depletion (STED) microscopy^5–7^, and stochastic optical reconstruction microscopy (STORM)^8–10^ have already demonstrated the use of ExM to increase the resolution of super-resolution methods. However, resolution alone is not sufficient for determining structure; equally important is the accuracy with which the structural measurements can be made^11^. While it remains unknown how accurately ExM preserves the structural features of the original samples on a scale of tens of nanometers and below, some evidence points to a lower limit for the scale at which structure can be preserved during ExM procedures. ExM typically relies on sodium polyacrylate/polyacrylamide gel (PAAG), which is a polyacrylamide (PA) based gel with added acrylate groups in order to enable expansion. PA-based gels are assembled through chain-growth radical chemistry polymerization, which due to the stochastic nature of the process introduces structural heterogeneities. Local PA gel defects in the form of loops and dangling ends can cause inhomogeneities on the 1–10 nm scale^12,13^, and variations in the polymer composition can lead to larger-scale defects. A small-angle X-ray scattering study found that these latter inhomogeneities were typically on the scale of 10–25 nm^14^, thus bringing into question whether PA-based gels have the ability to preserve structure on the scale of tens of nanometers.

**Figure 1:**
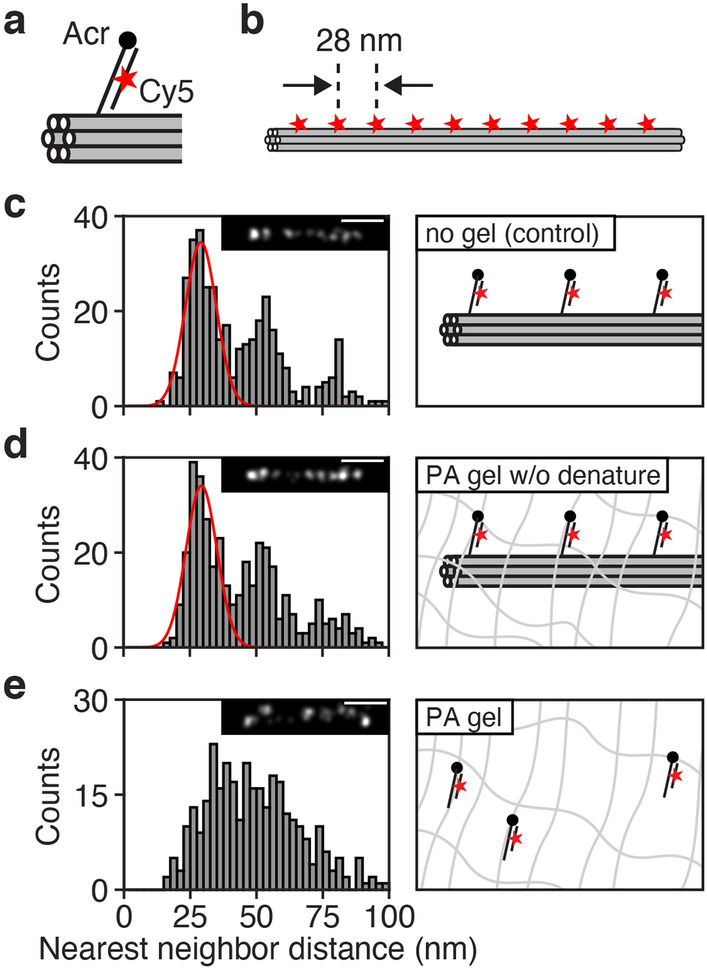
PA gel embedding does not preserve sub-30 nm structure. (**a**) DNA origami labeling strategy: Selected staple strands contain an acrydite group for PA gel anchoring and can later be fluorescently labeled through hybridization of a secondary oligo featuring an internal Cy5 dye. (**b**) Linear DNA origami design featuring 10 labeling sites spaced 28 nm apart. (**c–e**) STORM image analysis for linear origami (**c**) without gel (N = 100 origami objects), (**d**) post-embedding in a PA gel without denaturation (N = 100 origami objects), and (**e**) post-embedding in a PA gel (N = 98 origami objects). Insets show representative STORM images of single origami objects. The scale bars in the insets are 100 nm. Histograms show nearest neighbor emitter distances within origami objects. Red curves show Gaussian fits of the first peaks, mean ± standard deviation: (**c**) 29.1 ± 8.3 nm and (**d**) 29.4 ± 8.3 nm.

To quantify structural preservation in PA gels, we designed DNA origami^15^ nanorulers with precisely spaced labeling sites^16^ such that each site could be crosslinked to a gel and then localized using STORM. Each labeling site contained an acrydite group enabling crosslinking to a PA gel, as well as a 16-nt segment of single-stranded DNA (ssDNA) for hybridization to a DNA probe containing a single fluorescent dye (Fig. 1a). We used a linear 6-helix bundle design^17^ with 10 labeling sites spaced 28 nm apart (Fig. 1b), and imaged it with STORM, at first without gel embedding, as a reference (Fig. 1c, inset). In our STORM images, each photoswitchable Cy5 dye yielded multiple localizations spread in a cluster around the true location of the dye. We used an automated clustering algorithm (see “Methods”) to determine the center of each such cluster and were thereby able to determine the location of each fluorescent dye with an accuracy of a few nanometers. We then used these dye location data to construct a nearest neighbor distance distribution (Fig. 1c), to determine the consistency in the dye spacings. The nearest neighbor distance distribution showed a sharp peak centered around 28 nm, reflecting the designed spacings of labeling sites in the linear origami. The distribution also revealed additional peaks at multiples of 28 nm, likely reflecting cases where one or more emitters were missing. We used the location and width of the 28-nm peak as a reference for studying the effects of gel embedding.

To investigate whether the 28-nm spacings would be preserved after PA gel embedding, we anchored the origami labeling sites into a PA gel (without the expansion-enabling acrylate groups), at first without denaturing the origami. After casting the gel, we hybridized to each label a probe containing a fluorescent Cy5 dye, and then imaged the origami using STORM. The resulting nearest neighbor distance distribution (Fig. 1d) still showed a distinct 28-nm peak, demonstrating that the gelation process had not caused any major disruption of the original structure. However, in order to perform ExM, the original structure has to be disintegrated. We therefore repeated the previous measurement after soaking the gel in a solution containing formamide, which destabilizes double-stranded DNA and denatures the origami. Strikingly, the nearest neighbor distance distribution did not show a clear peak at 28 nm post denaturation (Fig. 1e), indicating that in the absence of the original structure, the original positions of the labels had not been retained in the PA gel.

We investigated several potential reasons for the discrepancy between the PA-embedded origami images with and without denaturing. First, we hypothesized that denaturation of the origami could have led to a reorientation of the flexible linkers between the acrydites and origami, and thereby in the linkers between the acrydites and the Cy5 dyes. In this way, even if the acrydites had remained in place during the embedding, the Cy5 positions could have moved, which in turn could have increased the variation of the nearest neighbor distances. To test this hypothesis, we conducted experiments using secondary probes designed to place the Cy5 dye immediately adjacent to the acrydite, meaning that the Cy5 emitter locations would accurately represent the positions of the acrydites and thus eliminating any effects of linker disordering. In these experiments, the nearest neighbor distances still showed clear discrepancy before and after denaturing (Extended Data Fig. 1), indicating that reorientation of the linkers alone had not caused the discrepancy. Rather, the acrydites must have moved from their original locations upon denaturation.

Second, we hypothesized that the denaturation of the origami might have created a void in the gel that might in turn have caused the gel during equilibration to warp, or otherwise change its local structure. To test this hypothesis, we performed experiments where we only dissociated the acrydite from the origami without denaturing the rest of the origami. We did this by attaching the acrydite to the origami via an oligo that could be dissociated from the origami by using toehold mediated strand displacement (Extended Data Fig. 2a). After anchoring the acrydites into the PA gel, we were thus able to dissociate the acrydite-oligo from the origami without the need to disintegrate the origami. The results from these experiments showed results similar to Fig. 1e, indicating that the acrydites had moved due to dissociation from the origami, rather than due to the void in the gel from origami denaturation (Extended Data Fig. 2b). Taken together, our results show that the PA gel did not accurately preserve 28-nm structural features.

**Figure 2:**
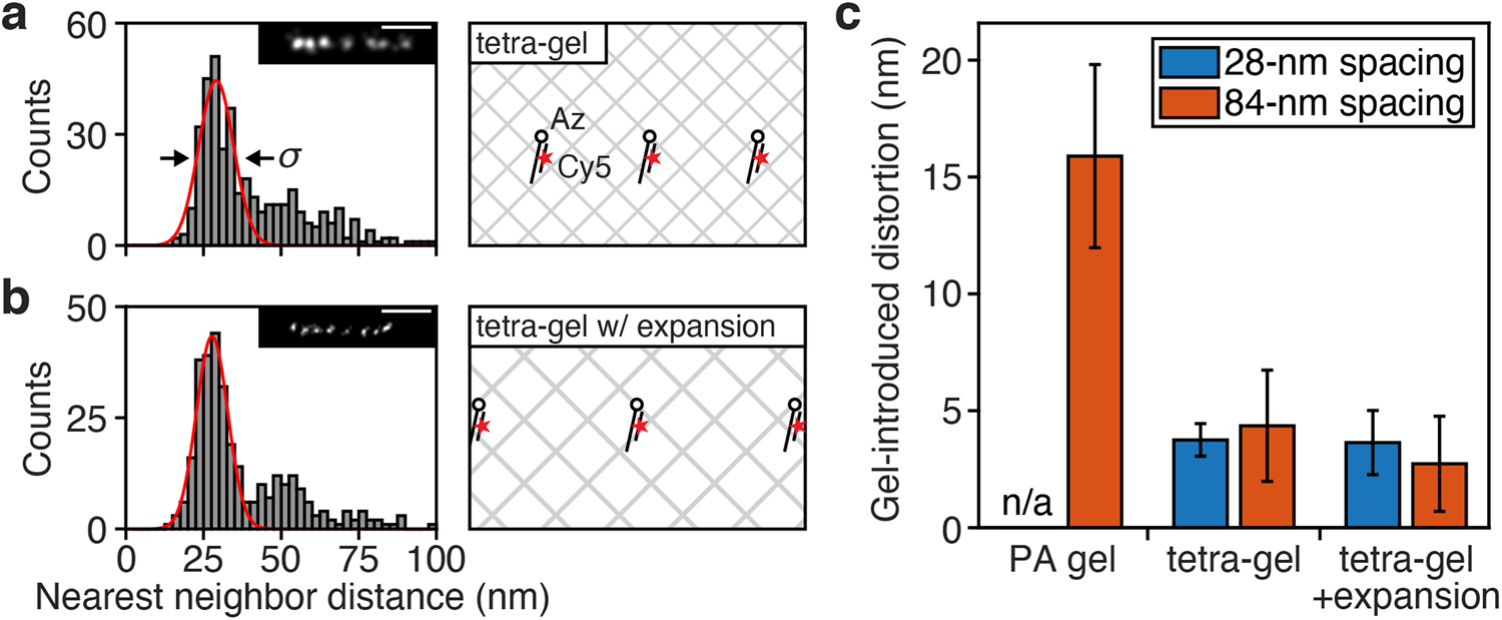
Tetra-gel embedding enables superior structural preservation at sub-10 nm scale. (**a**,**b**) STORM image analysis for linear origami with azides post-embedding in (a) non-expanding tetra-gel (N = 100 origami objects), and (**b**) expanding tetra-gel with 2.2X expansion (N = 83 origami objects). Insets show representative STORM images of single origami objects. The scale bars in the insets are 100 nm. Red curves show Gaussian fits of the first peaks, mean ± standard deviation: (**a**) 29.1 ± 8.0 nm and (**b**) 27.7 ± 7.2 nm; arrows indicate standard deviation *σ*. (**c**) Gel-introduced distortion in emitter position, calculated by comparing the standard deviations of individual emitter positions before and after denaturation in each gel (Extended Data Fig. 4). Bars and error bars show mean ± standard deviation.

To address the distortion associated with PA gel embedding, we set out to find an alternative strategy for gel embedding that would allow for improved structural preservation. A recent study reported the synthesis of tetra-gel, a new hydrogel compatible with expansion microscopy and featuring a higher structural homogeneity as compared with PA-based gels^18^. Tetra-gel is formed through terminal linking of two complementary tetrahedral polyethylene glycol monomers to create a diamond lattice-like polymer network. Importantly, tetra-gel monomers are linked with click chemistry, circumventing the need for free radical chemistry. To investigate whether tetra-gel embedding would preserve 28-nm structural features, we used the linear origami as described above, but replaced the acrydite anchors with azide groups since the tetra-gel relies on click chemistry. In sharp contrast to our PA gel results, we found that the 28-nm nearest neighbor distance peak was preserved in non-expanding tetra-gel even after origami denaturation (Fig. 2a), thus potentially enabling the combination of STORM with tetra-gel expansion without incurring the loss of accuracy seen with PA-based gels. When imaging the origami sample in expanding tetra-gel after expansion, we found that the fluorescent emitters were spaced farther apart, showing a higher resolution in accordance with the expansion factor of 2.2–2.5 as determined by measuring the physical size of the gel. Importantly, the nearest neighbor distance distribution still showed a sharp peak at 28 nm (scaled for expansion factor; Fig. 2b), indicating that the structural features of the sample had been preserved.

We then used our imaging data to quantify the distortions introduced by the different gels. To do this, we first measured the standard deviation of distance measurements (see *σ* in Fig. 2a; calculated from a Gaussian fit). We reasoned that *σ* would contain contributions from gel-induced distortions as well as contributions from intrinsic variations in sample structure. Noting that the distortion only appeared after denaturation of the origami structure, we could exclude non-gel contributions by comparing *σ* measured before and after denaturation. However, we were unable to accurately measure *σ* in the PA gel after denaturation (Fig. 1e), since the 28-nm peak was broadened to such an extent that it overlapped with higher-order peaks and thus precluded reliable fitting to a Gaussian. To circumvent this issue, we assembled a new linear DNA origami sample with identical structure, but with a labeling spacing of 84 nm instead of 28 nm. In this new sample with sparser labels, the nearest neighbor peaks no longer overlapped (Extended Data Fig. 3). We were thus able to determine *σ* under all examined conditions (Extended Data Fig. 4). We then measured the distortions introduced by the different gels, by comparing *σ* before and after denaturation, and used this information to calculate the average positional error for individual emitter locations (Fig. 2c). For the PA gel, we found a distortion of 16 nm, which is equivalent to 38 nm in full width at half maximum (FWHM). In contrast, the distortions in the non-expanding and expanded tetra-gels were all < 5 nm (< 12 nm FWHM), showing superior structural preservation. These results indicate that the combination of tetra-gel ExM with STORM could provide an increase in resolution without the loss of accuracy seen with PA-based gels.

**Figure 3:**
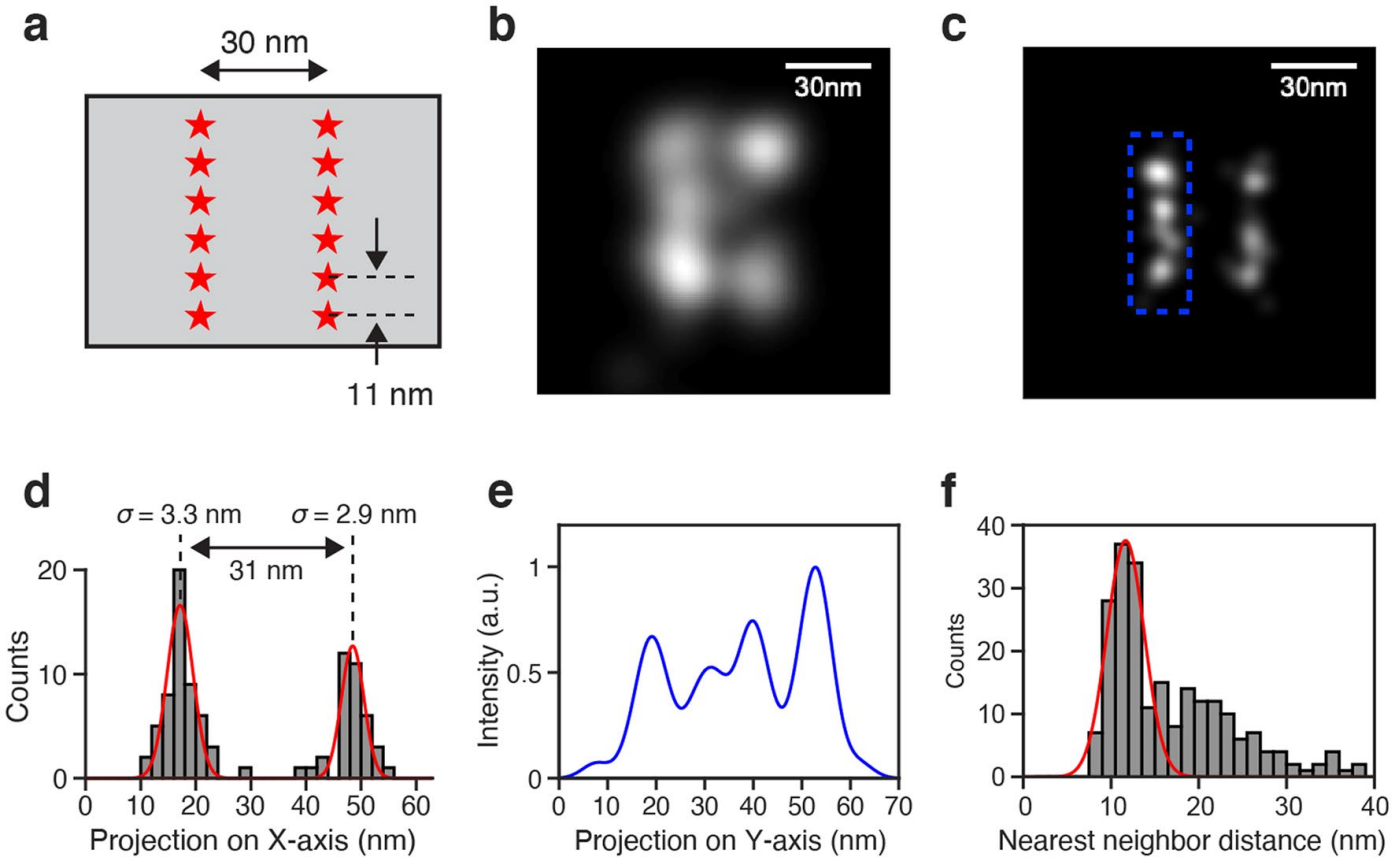
Tetra-gel expansion microscopy enables increased STORM resolution while preserving original structure. (**a**) Rectangular DNA origami design featuring labeling sites spaced 11 nm apart, organized in two rows spaced 30 nm apart. (**b**) Representative STORM image of a single rectangular origami object without gel. (**c**) Representative STORM image of a single rectangular origami object in tetra-gel, expanded 2.5X. (**d**) Projection of STORM localizations onto X-axis from the image shown in (**c**). Red curves show Gaussian fits. (**e**) Projection of blue area of image in **c** onto Y-axis. (**f**) Histogram of nearest neighbor emitter distances on the individual rows within rectangular origami objects (N = 93 rows). Post-expansion distances represent effective distances after scaling for the expansion factor. Red curve shows Gaussian fit of the first peak, mean ± standard deviation: 11.7 ± 3.0 nm.

Next, we set out to explore the potential for using tetra-gel ExM to increase the resolution of STORM. For these measurements, we constructed a new DNA-origami reference sample with the intention of minimizing intrinsic sample variation from structural warping. To restrict warping, we used a flat rectangular origami design^19,20^ with multiple biotin attachment points spread out in two dimensions. We reasoned that when tethered to a surface, these dispersed biotin linkages would restrict deformation of the structure, since any warping would require out-of-plane movements (unlike the linear origami which could warp by bending in the image plane). The rectangular origami featured labeling sites arranged in two rows spaced 30 nm apart (Fig. 3a). Within each row, the individual labeling sites were spaced 11 nm apart. In STORM images of the sample without gel embedding, we could clearly detect the two rows, however the 11-nm spacings were not readily resolved (Fig. 3b, Extended Data Fig. 5a). STORM images of samples expanded using tetra-gel ExM showed well-resolved individual labeling sites as well as a preserved overall structure (Fig. 3c, Extended Data Fig. 5b).

Analysis of a single rectangular origami object imaged with STORM post tetra-gel ExM revealed a well-preserved row spacing and low deviation from straightness within the individual rows (Fig. 3c). The standard deviation of emitter localizations in each row were around 3 nm along the perpendicular axis (Fig. 3d) and when we projected one of the rows onto a parallel axis, the projected pattern showed clearly resolved peaks spaced ~ 11 nm apart (Fig. 3e). Using data from a large number of origami objects in three separate experiments, we constructed nearest neighbor distance histograms for fluorescent maxima along individual rows and found that the histograms showed sharp peaks centered at ~ 11 nm and with a standard deviation of 3 ± 1 nm (Fig. 3f). These results show that the addition of tetra-gel expansion led to an increase in resolution while maintaining high accuracy.

In summary, we found that PA gel embedding does not preserve molecular structure sufficiently to allow for accurate structural determination below 30 nm. We further show that this obstacle can be overcome by using new gel chemistries. In particular, our results demonstrate that a recently developed tetra-gel allows for greater structural preservation during embedding and expansion, to the point where the addition of tetra-gel expansion to STORM enables a higher determination of structural detail in the original sample. Our work thus opens the door to combined ExM-super-resolution investigations with confidence in structural preservation.

## Methods

### DNA origami preparation and purification

Linear and rectangular DNA origami were designed using caDNAno and prepared as described previously^21^, with added modifications for surface attachment and labeling (see Supplementary Tables). All DNA oligomers, including origami staple strands and additional DNA linkers, were ordered from Integrated DNA Technologies (IDT). All origami structures contained extension strands with single-stranded DNA (ssDNA) overhangs (“handles”) for hybridization to additional ssDNA oligos. Primary, secondary, and tertiary staple strands were ordered with HPLC purification. DNA modifications are indicated using their IDT codes. For attachment of the DNA origami objects to a streptavidin-coated coverslip, we incorporated 4 strands with binding sites for the biotinylated oligomer (‘Hr21_5Bio’) in the linear structures^17^, and 8 strands directly modified with biotins in the rectangular structures^19,20^.

DNA staple strands and the M13mp18 viral DNA (New England BioLabs) used as the scaffold were mixed in folding buffer: 10 mM Tris, pH 8.0, 1 mM EDTA, and 18 or 15 mM MgCl_2_ for the linear and rectangular structures, respectively. The concentrations of DNA were 10 nM for the scaffold strand, 100 nM for the unlabeled staple strands, 0.5 µM for the labeled primary staple strands, and 4 µM for the secondary staple strands for each labeling site. The origami mixtures were incubated and annealed using a thermocycler. For the linear origami, these mixtures were held at 80 °C for 5 min to remove any residual structure, and annealed by cooling, first to 65 °C in 1 °C steps every 5 min, then to 25 °C in 1 °C steps every 40 min. The rectangular origami was folded by heating to 80 °C for 5 min, and annealed by cooling, first to 65 °C in 1 °C steps every 5 min, then to 25 °C in 1 °C steps every 20 min. The origami samples were then purified by agarose gel electrophoresis. Electrophoresis was performed with a 2% agarose gel in an ice bath in running buffer containing 89 mM Tris, 89 mM borate, 2 mM EDTA, and 10 mM MgCl_2_. The origami band, visualized using SYBR Safe (Thermo Fisher) and blue light illumination, was excised from the gel and the origami was then extracted using a Freeze ‘N Squeeze spin column (Bio-Rad) by spinning at 1000 g for 60 min.

### Preparation of coverslips, gel embedding, sample denaturation, and gel expansion

The origami control samples without any gel were imaged in a flow chamber consisting of a glass coverslip (No. 1.5; VWR) attached to a microscope slide with double sided tape. The coverslip was pre-coated with biotinylated BSA (Thermo Fisher) and streptavidin (New England BioLabs), and the biotin-containing origami sample was then flown onto the coverslips. Then the flow chamber was filled with imaging buffer and both of its sides were sealed with nail polish (Electron Microscopy Sciences).

To stabilize the gel on the coverslip, the coverslips for non-expanding gels of both PA gel and tetra-gel were silanized as published previously^22^. The PA gel solution consisted of 2.5% (vol/vol) of 19:1 acrylamide/bis-acrylamide (Bio-Rad), 60 mM Tris–HCl pH 8, 10 mM MgCl_2_, 2 M NaCl, and 0.2% (vol/vol) Tetramethylethylenediamine (TEMED; Sigma-Aldrich). The polyacrylamide solution was kept on ice and further supplemented with ammonium persulfate (APS; Sigma-Aldrich) at a final concentration of 0.2% (wt/vol). The coverslips for the origami with PA gel were then treated with biotinylated BSA and streptavidin and finally coated with origami. The de-gassed gel solution was added to the surface of a glass plate that had been pretreated for 5 min with 1 mL GelSlick (Lonza) so as not to stick to the polymerized gel. Coverslips containing samples were aspirated, dried quickly with KimWipes (Kimberly-Clark) from the edge of the coverslips, and gently inverted onto the gel droplet to form a thin layer of solution between the coverslip and the glass plate. The gel solution was then allowed to polymerize in a nitrogen chamber at room temperature for 2 h.

The expanding tetra-gel was made as published previously^18^. In the earlier study describing the tetra-gel, two chemical groups were suggested for clicking to azides: DBCO and BCN. While using DBCO appeared to negatively affect the switching properties of Cy5, we did not observe the same effect when using a BCN-based tetra-gel. All of the tetra-gel results presented here were therefore obtained using the BCN version of the gel with 10 mM MgCl_2_. To make a non-expanding tetra-gel, we replaced “monomer 1” with 4-Arm PEG-Azide, MW 10 k (Creative PEGWorks) at the same concentration. With 14% DMSO in the tetra-gel, an extra step of fixation was needed; we found that otherwise the origami would not tether properly to the coverslip, presumably due to DMSO-induced detachment or denaturation of the streptavidin. For the extra fixation step, the coverslips were treated with 2% glutaraldehyde (GA) in PBS for 10 min, and then with 0.1% (wt/vol) sodium borohydride in PBS for 7 min. Finally, the coverslips were washed by PBS three times before adding the origami to the coverslip. After the origami were attached to the coverslips, the coverslip was inverted onto the tetra-gel solution and left in a humidified chamber at room temperature overnight.

The origami in the gel were denatured by incubating in 60% formamide and 2 M NaCl for 1 h at room temperature. The expanding gels were then transferred into petri dishes and expanded in water. The water was replaced three times, at 30-min intervals.

### Post-expansion gel re-embedding

The non-expanding re-embedding gel solution consisted of 4% (vol/vol) 19:1 acrylamide/bis-acrylamide, 0.0005% (wt/vol) riboflavin 5′-monophosphate (for photopolymerization; Fisher Scientific), 0.05% (vol/vol) TEMED, and 0.0075% (wt/vol) APS. The solution was de-gassed for 5 min prior to usage. The original gels were incubated in 3 mL of the re-embedding gel solution for 60 min at 4C in the dark. 200 µL of the re-embedding gel solution was added to the surface of a glass plate that had been pretreated for 5 min with 1 mL GelSlick. The expanded gels were then transferred onto silanized coverslips and the excess re-embedding gel solution was removed. Samples were then gently inverted onto the 200-μL droplet of re-embedding gel solution on the glass plate. The samples the were then illuminated by ultraviolet (UV) light at 12 mJ/cm^2^ for 2 h to polymerize in a humidified chamber.

Gel expansion is driven by a change in the immersion solution’s ionic strength; a lower final salt concentration leads to a higher expansion factor. Conversely, any addition of salt to the immersion solution after expansion results in a reduction of the expansion factor. Our imaging buffer, however, contained an oxygen scavenging system relying on salt to prevent acidification during imaging. This led us to seek a way to stabilize the tetra-gel post expansion but prior to imaging, in order to prevent sample drift and shrinkage. Other works have previously reduced the drift of expansion gels during imaging either by adhering the gels to the coverslips with lysine-coating or low-melting point agarose^3,5,8,9,23–27^, or by re-embedding the gel in a second PA gel to fix the entire sample^10,22,25^. We found that immobilizing the expansion gel on the coverslips did not prevent shrinkage once our imaging buffer was introduced. We therefore opted to re-embed the expansion gel in a PA gel after expansion. This strategy also presented a challenge, however, since initiation of the PA polymerization reaction typically requires an initiator such as ammonium persulfate (APS), which is highly charged and thus would also reduce the expansion factor of the tetra-gel. At the concentration of APS (0.05% wt/vol) required to cast the PA gel for re-embedding, we found that the tetra-gel expansion factor was reduced by ~ 30%. To overcome this challenge, we used UV light to photochemically initiate the acrylamide polymerization reaction, which allowed us to use a lower concentration of APS. With UV irradiation, we were able to initiate polymerization of the PA gel using only 0.0075% APS (wt/vol). Under these conditions, we achieved a final tetra-gel expansion factor of 2.2–2.5 as determined by measuring the physical size of the gel, including less than 10% of shrinkage upon re-embedding. After re-embedding, we did not observe any remaining sensitivity of the gel size to variations in solution ionic strength.

### Fluorescent labeling

All samples featuring origami in a gel were incubated in 2X Saline-Sodium Citrate (SSC) buffer (Thermo Fisher) for 1 h before the hybridization. The non-expanding PA gel samples were hybridized with DNA labels with Cy5 (IDT). The other samples were hybridized with Locked Nucleic Acid (LNA) oligonucleotides (IDT) after an additional blocking step by incubating in blocking buffer consisting of 0.1X SuperBlock (Thermo Fisher), 30% formamide (Thermo Fisher), and 2X SSC for 30 min. The samples were incubated in 30% formamide, 2X SSC, and 100 nM of dye labeled oligos for hybridization for 30 min. Finally, the samples were washed with 30% formamide and 2X SSC for 30 min.

### STORM imaging

The imaging buffer was based on 2X SSC and contained 120 mM cysteamine (Sigma-Aldrich), 10% glucose, 50 mM Tris–HCl, 3.8 mg/mL glucose oxidase (Sigma-Aldrich), and 240 μg/mL catalase (Sigma-Aldrich). Approximately 2 mL of imaging buffer was injected into the flow chamber for imaging.

Imaging was performed through a 60× 1.4 NA oil-immersion objective (Nikon) mounted on an Eclipse Ti inverted microscope (Nikon) with back optics arranged for oblique incident angle illumination. The microscope contained a custom pentaband dichroic (Chroma Technology) and pentanotch filter (Chroma Technology) and a 647 nm laser (MPB Communications) for excitation of Cy5. A 405 nm laser (Coherent) was used for reactivation of dyes. Images were acquired on a CMOS camera (Hamamatsu, Orca Flash 4.0v2) at 25 Hz frame rate. Each camera pixel corresponded to 153 nm in sample space, and the total imaging field size was ∼90 μm × 90 μm. Approximately 15,000 frames were recorded to reconstruct each STORM image. Axial focus during imaging was maintained in an automated manner as described previously^28^.

### Image analysis

The centroid positions of the single-molecule images provided lateral positions of each activated fluorescent molecule. The final super-resolution images were reconstructed from these molecular coordinates by depicting each location as a 2D Gaussian peak with 8-nm rendering radius. To determine the clusters of localizations corresponding to each emitter, the DBSCAN algorithm was performed on the collected centroid positions of localizations. The parameters of DBSCAN (eps = 10–15 nm, minPts = 5) were set to match the imaging properties of Cy5 dyes, hence the centroid positions of each of the clusters should accurately report the positions of the corresponding emitters. Images of linear origami objects were selected if the object contained 5 or more clusters in a straight line, as defined by a linear regression of the clusters having an R-squared above 0.9. We note that our selection may have excluded objects with high distortion in the transverse direction, which in turn may have led to a slight underestimation of the distortion. The nearest neighbor emitter distances in Figs. 1 and 2 were calculated based on the Euclidean distances between the DBSCAN cluster centers. To ensure each distance was only counted once, all of the duplicates of the distances within the same origami were removed. In the case of the 28 nm-spacing origami in the PA gel after denaturation, the distortion was such that in some cases pairs of localization clusters became spaced sufficiently close together to be misinterpreted as a single cluster. The true spacing distance between these erroneously merged clusters would be missing from the corresponding nearest neighbor distance histogram, thus contributing to a suppression of counts on the lower end of the histogram. Another consequence of this erroneous merging of clusters is that the new (erroneous) cluster center would be situated farther from the nearest neighboring clusters on either side, thus contributing to an overall inflation of the measured nearest neighbor distances.

For the rectangular origami, we first selected the regions of interest with more than 30 localizations within 4 μm^2^ area. Then we fitted two lines to each image to identify the two rows on each rectangular origami. DBSCAN did not work well for clustering the rectangular origami with 10-nm spacing, because the DBSCAN would incorrectly join clusters if they were too closely spaced. Instead, we found the local maxima in Gaussian blurred rectangular origami images, and further assigned these local maxima as the initial guesses for clusters to perform K-means clustering on the localizations. The centroid positions of these clusters were then determined based on the results of the K-means clustering. Rows on the rectangular origami images were selected if there were 3 or more clusters in a straight line, as defined above. Nearest neighbor emitter distances were then calculated as for the linear origami.

## Supplementary Information

Supplementary Figures.

Supplementary Tables.
