# Supplementary material for: Tetra-gel enables superior accuracy in combined super-resolution imaging and expansion microscopy
Source: Sci Rep. 2021 Aug 20;11:16944. doi: 10.1038/s41598-021-96258-y (PMC8379153; doi:10.1038/s41598-021-96258-y)
Supplement: Supplementary file 1 — Supplementary Figures. [file 41598_2021_96258_MOESM1_ESM.pdf]

Supplementary Information

Tetra-gel enables superior accuracy in combined super-resolution imaging and expansion microscopy

Hsuan Lee, Chih-Chieh Yu, Edward S. Boyden, Xiaowei Zhuang, Pallav Kosuri

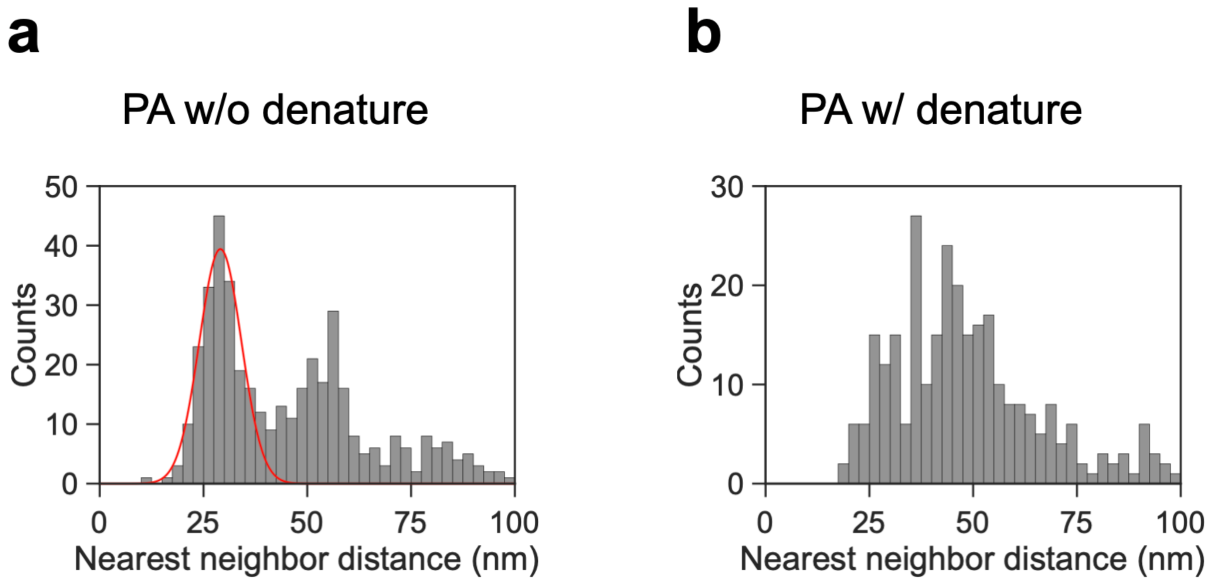

Extended Data Fig. 1 | The linkers between acrydites on primary handles and Cy5 dyes on secondary probes do not account for the distortion in PA gel. STORM image analysis of the 28-nm spacing linear origami hybridized with secondary probes whose Cy5 dyes were placed on the 3' end and thus immediately adjacent to the acrydites, **a**, post-embedding in a PA gel without denaturation (N = 111 origami objects), and **b**, post-embedding in a PA gel and after denaturation of the original structure (N = 84 origami objects). Histograms show nearest neighbor emitter distances within origami objects. Red curve in **a** shows Gaussian fit to the first peak, mean ± standard deviation: 29.1 ± 7.2 nm

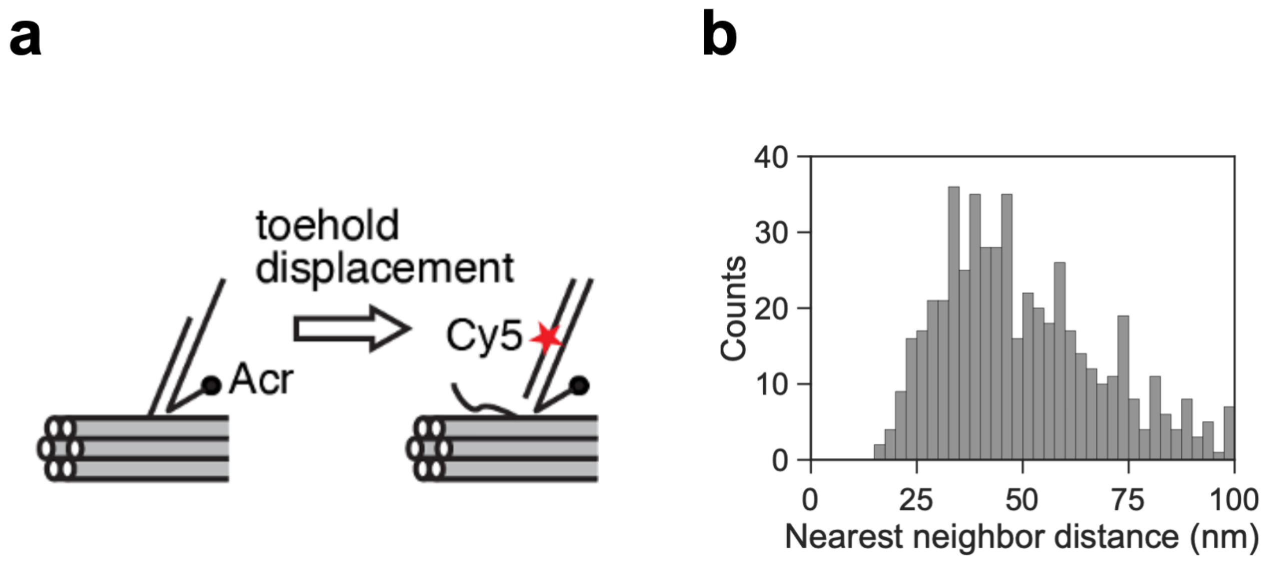

Extended Data Fig. 2 | PA gel embedding does not preserve 28 nm spacing even without removal of the origami structure. **a**, DNA origami labeling strategy. To test whether the denaturation protocol had caused the distortion of the origami, we placed the acrydite on an oligo hybridized to the 28-nm spacing origami (as opposed to an oligo directly incorporated in the origami). The acrydite-oligo could then later be fluorescently labeled through toehold displacement, which we reasoned would dissociate the acrydite-oligo from the origami without denaturing the origami. To verify that the toehold displacement procedure did not disrupt the origami structure, we subjected our original, Cy5-labeled origami without acrydite to the toehold hybridization condition and found that its structure remained intact. **b**, Nearest neighbor emitter distance histogram from STORM images of linear origami with 28-nm spacing, post-embedding in a PA gel and fluorescently labeled using toehold displacement without denaturation of the original structure (N = 155 origami objects). Similar to the histogram in **Fig. 1e**, no sharp peak can be seen in this histogram. These results indicate that the denaturation of the origami had not caused disordering of the PA-anchored acrydites in **Fig. 1e**. Rather, it appears that the acrydites' connection to the origami had prevented them from moving. The acrydites then became disordered once they were disconnected from the origami, either through origami denaturation or toehold displacement.

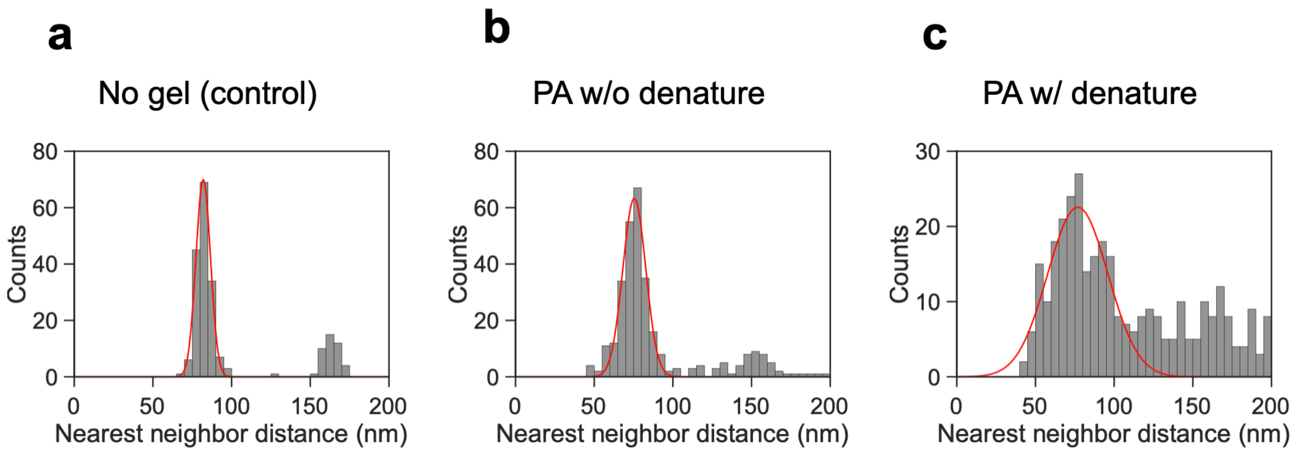

Extended Data Fig. 3 | PA gel embedding distance measurement on linear origami structure with 84-nm spacing between labels. **a-c,** STORM image analysis for 84-nm spacing linear origami (**a**) without gel (N = 100 origami objects), (**b**) post-embedding in a PA gel (N = 152 origami objects), and (**c**) post-embedding in a PA gel and after denaturation of the original structure (N = 167 origami objects). Histograms show nearest neighbor emitter distances within origami objects. Red curves show Gaussian fits to the first peaks, mean ± standard deviation: (**a**) 82.0 ± 6.5 nm, (**b**) 75.5 ± 10.3 nm, and (**c**) 76.9 ± 27.1 nm. Under all gel-embedded conditions, standard deviations in distance were consistently larger for the 84-nm samples (around 12 nm) than for the 28-nm samples (around 8 nm). This was likely due to some of the origami objects becoming tilted away from the imaging plane during gel embedding. We concluded this for the following reasons: First, if the origami were tilted away from the imaging plane, then we would observe a decrease in the mean spacing distance. Indeed, we observed a ~7% decrease in the center location of the first peaks in the nearest neighbor distance histograms in **b** and **c**, compared with the no-gel control case in **a**. We would expect these effects to be less noticeable in the 28-nm sample, due in part to the shorter baseline distance, and in part to the potential of objects with smaller emitter spacings to be screened out by the clustering algorithm due to cluster overlap.

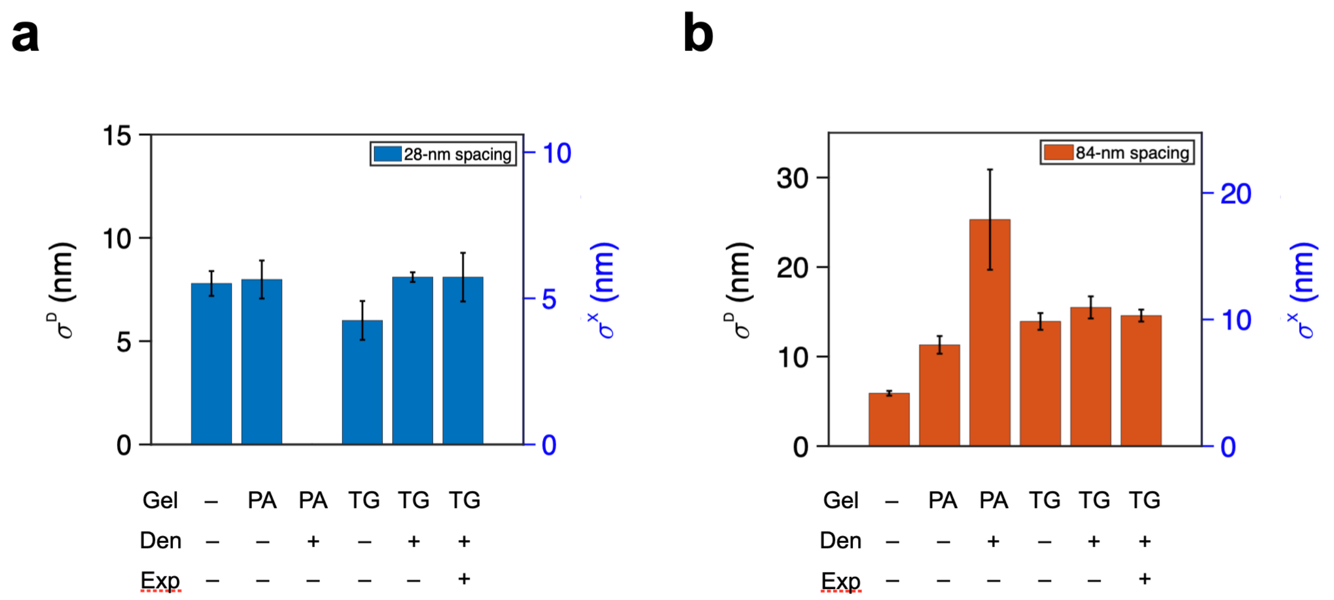

Extended Data Fig. 4 | a-b, Standard deviations of nearest neighbor distances in (**a**) 28-nm and (**b**) 84-nm spacing origami in different conditions. Values were measured as the standard deviation of a Gaussian fit to the first peak in nearest neighbor distance histograms. For the 28-nm origami data in the PA gel, we were unable to reliably fit a Gaussian to the first peak due to overlap with the neighboring peak. Right axes: Standard deviations of individual emitter positions. We define the distance between two individual emitters as D = |X-Y|, then the variance of the distance is Var(D) = Var(X) + Var(Y) – Cov(X,Y). If we assume that the variations in emitter positions are independent of one another [Cov(X,Y) = 0], and since X and Y are drawn from the same population of emitters [Var(X) = Var(Y)], we get Var(D) = 2*Var(X). More details can be found in the reference[11]. The standard deviations in distances were measured from Gaussian fits to the nearest neighbor histograms, which is $\sigma^D = \sigma^X * \mathrm{sqrt}(2)$, hence the standard deviation of the individual emitter positions is $\sigma^X = \sigma^D / \mathrm{sqrt}(2)$. Data from three independent experiments for each condition (N > 60 origami objects in each experiment). Bars and error bars show mean ± standard deviation. "Den" = after denaturation of original origami structure, "Exp" = after expansion, "PA" = polyacrylamide gel, "TG" = tetra-gel. We could then calculate the distortion introduced by the gel by comparing the standard deviations of individual emitter positions before and after denaturation in each gel. If we assume that all variations follow Gaussian distributions then we can calculate the distortion introduced by the gel as $\sigma_{\mathrm{gel}} = \mathrm{sqrt}[(\sigma^X_{\mathrm{w/\ denature}})^2 - (\sigma^X_{\mathrm{w/o\ denature}})^2]$. Measurement uncertainties were determined by error propagation. The results of these calculations are shown in **Fig. 2c**.

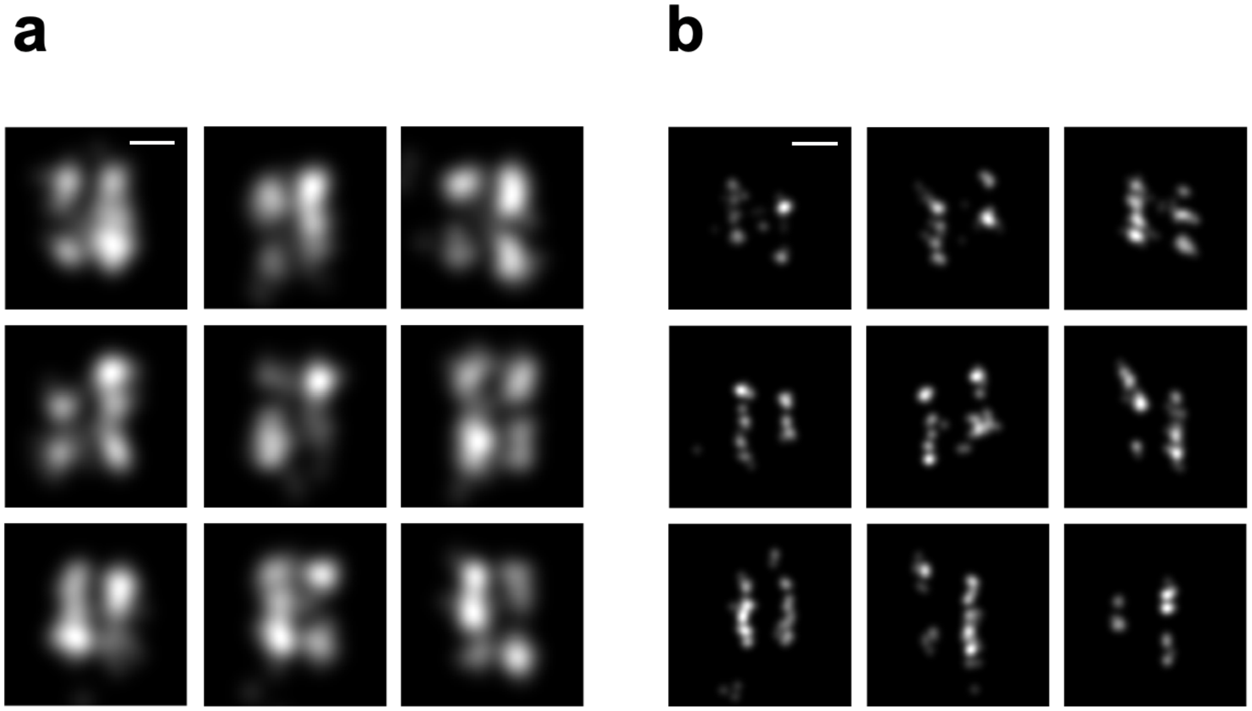

Extended Data Fig. 5 | a-b, STORM images of rectangular origami (**a**) without gel and (**b**) in a tetra-gel with 2.5X expansion. The scale bars are 30 nm.
